# The role of immune cells in different stages of atherosclerosis

**DOI:** 10.7150/ijms.94570

**Published:** 2024-04-22

**Authors:** Cong He, Hyo In Kim, Jinbong Park, Junli Guo, Wei Huang

**Affiliations:** 1 Key Laboratory of Tropical Translational Medicine of Ministry of Education, School of Basic Medicine and Life Sciences, Hainan Medical University, Haikou 571199, PR China.; 2 Key Laboratory of Tropical Translational Medicine of Ministry of Education & Key Laboratory of Tropical Cardiovascular Diseases Research of Hainan Province, School of Public Health, Hainan Medical University, Haikou 571199, PR China.; 3 Department of Pharmacology, Harbin Medical University-Daqing, Daqing 163319, PR China.; 4 Department of Surgery, Beth Israel Deaconess Medical Center, Boston, MA 02215, United States.; 5 Department of Pharmacology, College of Korean Medicine, Kyung Hee University, Seoul 02447, Republic of Korea.

**Keywords:** atherosclerosis, inflammation, innate immunity, adaptive immunity

## Abstract

Atherosclerosis is a chronic inflammatory disease characterized by the accumulation of immune cells in the intima of arteries. Experimental and clinical evidence shows that both innate and adaptive immunity orchestrate the progression of atherosclerosis. The heterogeneous nature of immune cells within atherosclerosis lesions is important. Studies utilizing high-dimensional mass spectrometry and single-cell RNA sequencing of leukocytes from atherosclerotic lesions show the diversity and adaptability of these immune cell subtypes. Their migration, compositional changes, phenotypic alterations, and adaptive responses are key features throughout atherosclerosis progression. Understanding how these immune cells and their subtypes affect atherogenesis would help to develop novel therapeutic approaches that control atherosclerosis progression. Precise targeting of specific immune system components involved in atherosclerosis, rather than broad suppression of the immune system with anti-inflammatory agents, can more accurately regulate the progress of atherosclerosis with fewer side effects. In this review, we cover the most recent advances in the field of atherosclerosis to understand the role of various immune cells on its development. We focus on the complex network of immune cells and the interaction between the innate immune system and adaptive immune system.

## Introduction

Cardiovascular diseases (CVDs) include myocardial infarction, heart failure, ischemic stroke, and sudden cardiac death. CVDs are the main causes of highly morbidity and mortality in both developed and developing countries 1. Atherosclerosis is the main cause of cardiovascular diseases. It is a chronic inflammatory disease which occurs in the intima of middle and large arteries 2. Both innate and adaptive immunity play important roles in the progression of atherosclerosis. Infiltrated low-density lipoprotein (LDL) can activate endothelial cells, and subsequently induce the recruitment of monocytes 3. Macrophages derived from monocytes absorb lipoproteins to form lipid rich foam cells, leading to the formation of necrotic cores and eventually forming atherosclerosis plaques 4. In addition, macrophages in plaques recognize LDL derived epitopes and release various pro-inflammatory mediators to drive the initiation of vascular inflammation 4. Furthermore, macrophages, along with dendritic cells (DCs), mediate antigen presentation in secondary lymphoid organs which accelerates excessive plaque growth through triggering an adaptive immune response 5. Advanced plaques are characterized by large necrotic cores containing activated immune cells, extracellular lipids and lipoproteins derived cholesterol crystals. These plaques are located under the fibrous cap composed of smooth muscle cells (SMCs) and collagen rich matrix, preventing them from interacting with platelets 6. Rupture or erosion of plaques may trigger thrombosis in the coronary arteries which directly cause myocardial infarction 7. In this review, we summarize literature on the role of the immune system in atherosclerosis progression, with particular attention to the interaction between innate and adaptive immunity in atherogenesis.

## Innate immunity

In the artery wall, LDL undergoes modification and acquires immunogenic properties triggering the initiation of innate immunity that attracts innate and adaptive immune cells 4. Monocytes and macrophages can polarize to pro-inflammatory phenotypes upon certain stimuli, then possess potentially pro-atherogenic roles 8. Dendritic cells and neutrophils aggregation, and cause arterial inflammation which drives the expansion of atherosclerotic lesions 9, 10. The role of other innate immune cells including mast cells and natural killer T (NKT) cells in atherosclerosis have been previously reviewed 11.

### Monocytes

**Figure 1:**
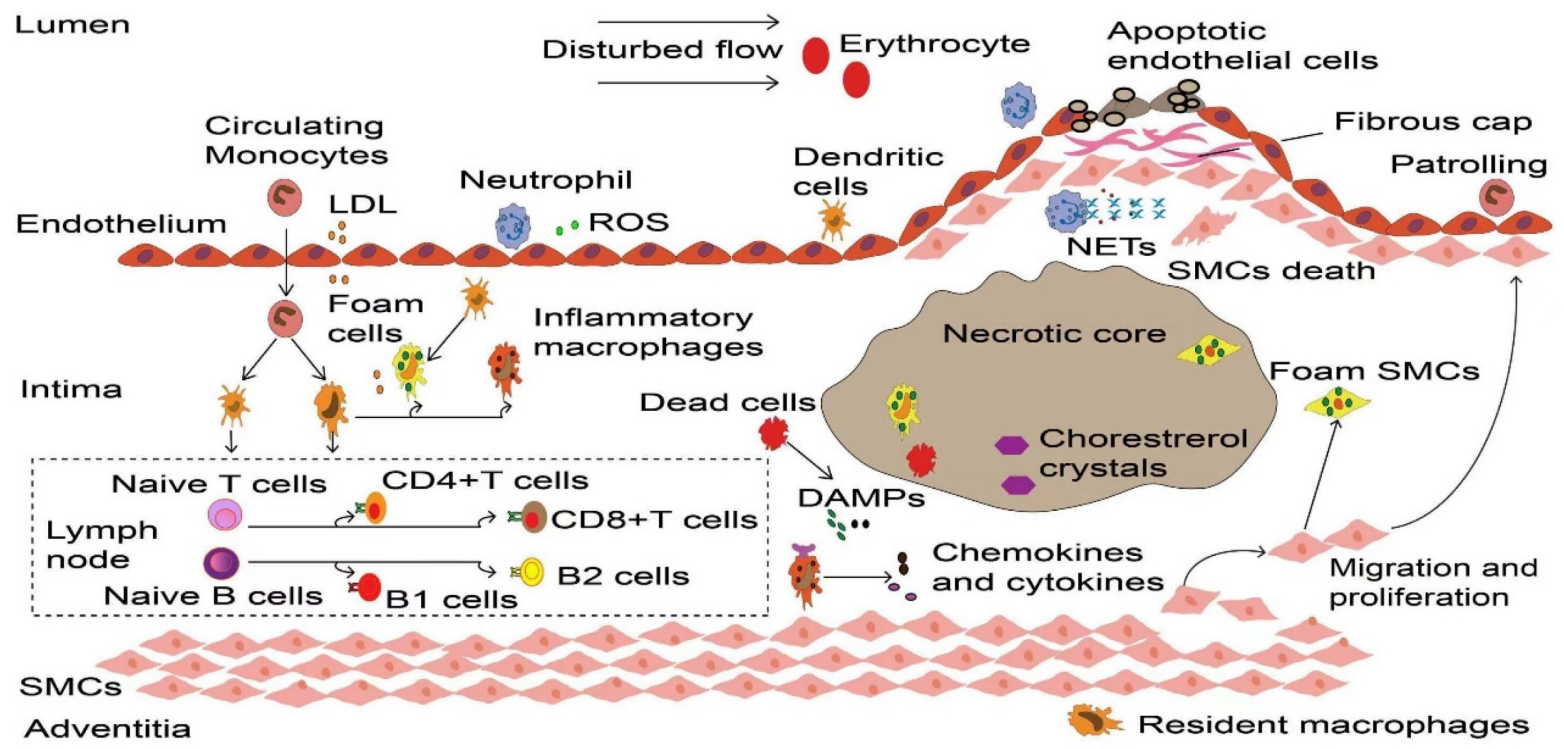
** Dynamic process of inflammatory cells in atherosclerosis.** Upon the vascular endothelial cell dysfunction induced by disturbed flow, erythrocytes and LDL enter the intima that drives the initiation of atherosclerosis. These atherogenic particles trigger vascular inflammation, thereby accelerate monocyte and neutrophil recruitment. Neutrophils release ROS and various granule proteins increase chemotactic molecules expression in activated endothelial cells, in turn attract more immune cells into intima. Monocyte-derived macrophages, partly intimal DCs and SMCs that acquire macrophage properties together take up LDL and become foam cells in the onset of atherosclerosis. Persistence of accumulated LDL leads to foam cells stress and death, these dead cells form necrotic cores and eventually develop atherosclerotic plaques. Inflammatory macrophages recognize ROS-induced oxLDL and DAMPs provided by dead cells, releasing various cytokines and chemokines to reconstruct the microenvironment in atherosclerosis. More importantly, DCs along with macrophages present LDL-derived antigen to naive B and T cells in artery tertiary lymphoid organs that located in the surrounding of diseased arteries such as the adventitia, lead to autoreactive B cells and T cells. These adaptive immune cells start proliferation and differentiation, producing cytokines and antibodies to accelerate or dampen atherosclerosis progression. To prevent plaque components interacting with platelets, SMCs transmigrates and their derived collagen matrix together constitute a fibrous cap that overlaps advanced plaque in sub-endothelial space. Meanwhile, neutrophils and their secondary products such as NETs promote the death of endothelial cells and SMCs, which elicit plaque rupture or erosion, respectively. LDL, low-density lipoprotein. ROS, Reactive oxygen species. DCs, dendritic cells. SMCs, smooth muscle cells. oxLDL, Oxidized low-density lipoprotein. DAMPs, damage-associated molecular patterns. NETs, neutrophil extracellular traps.

Monocytes are one of the key cells of the innate immune system. Monocytes are rarely seen in healthy coronary arteries. Atherosclerosis-related inflammation and hypercholesterolemia destroy the homeostasis of monocytes in blood, bone marrow and spleen 12. This promotes the maturation of hematopoietic stem cells and progenitor cells into monocytes 13. Circulating monocytes are positively correlated with the severity and plaque size of atherosclerosis 14, thus is a promising target for treatment. Inhibiting monocyte accumulation in the arterial intima almost eliminates atherosclerosis in mice 15. Monocytes are usually be divided into two categories: classical monocytes (Ly6C^high^ in mice and CD14+ CD16- in humans) and non-classical monocytes (Ly6C^low^ in mice and CD14^low^ CD16+ in humans) 14, 16. Classical monocytes accumulate in the intima of artery wall and are necessary for the initiation and progression of innate immunity 4. In particular, the C-C chemokine receptor type 2 (CCR2, CCR5) and C-X-(3)-C motif chemokine receptor 1(CX3CR1) of classical monocytes play an important role in development of atherosclerotic plaques 15. Plaque monocytes not only transform into macrophages but also produce DCs (Fig. 1), which together trigger local inflammation and thus accelerate the progression of atherosclerosis 17. Non-classical monocytes, which accounts for ~10% of the total monocyte population 18, function to maintain the vascular homeostasis without transmigration through the endothelium. Unlike classical monocytes, most non-classical monocytes have been widely viewed as anti-inflammatory, as maintain vascular homeostasis that patrol and crawl along arterial endothelium to resolve arterial inflammation 16, 19.

### Macrophages

Macrophages are the most abundant innate immune cells found in atherosclerosis 20. Macrophages predominate in all stages of atherosclerosis, including lesion initiation, foam cell formation, necrotic core expansion, plaque rupture or erosion 21, lesion regression and inflammation 22. Macrophages exist in adventitia and intima of healthy arteries 23. These resident macrophages originate from embryonic hematopoiesis, infiltrate before or shortly after birth, and are maintained through local proliferation 24. In general, resident macrophages can prevent arteriosclerosis by maintaining vascular homeostasis through interaction with vascular smooth muscle cells 25 and by eliminating apoptotic cells and damaged mitochondria 26. The outer membrane macrophages with high expression of lymphatic vessel endothelial hyaluronic acid receptor 1 (LYVE1) limit inadaptable cardiac remodeling after myocardial infarction 27. On the other hand, resident macrophages in the aortic intima can promote atherosclerosis during the early stage by acting as the earliest foam cell in the lesions 23.

Resident macrophages and monocyte-derived macrophages are bound by CCR2 on cell surface 26, which reconstructs macrophages in lesions and form the majority of macrophages through continuous infiltration of classical monocytes 15. Macrophages are the pivotal factor in the conversion of pathological intimal thickening into early atherosclerotic plaques by promoting necrotic core formation 28. Macrophages also capture and phagocytose modified LDL through scavenger receptors such as scavenger receptor type A (SR-A) and cluster of differentiation 36 (CD36) 29. Continuous engulfment of lipoprotein particles matures macrophage into foam cells 21. Even more, SMCs accelerate plaque formation after turning into macrophage-like cells (Fig. 1) 30.

**Figure 2:**
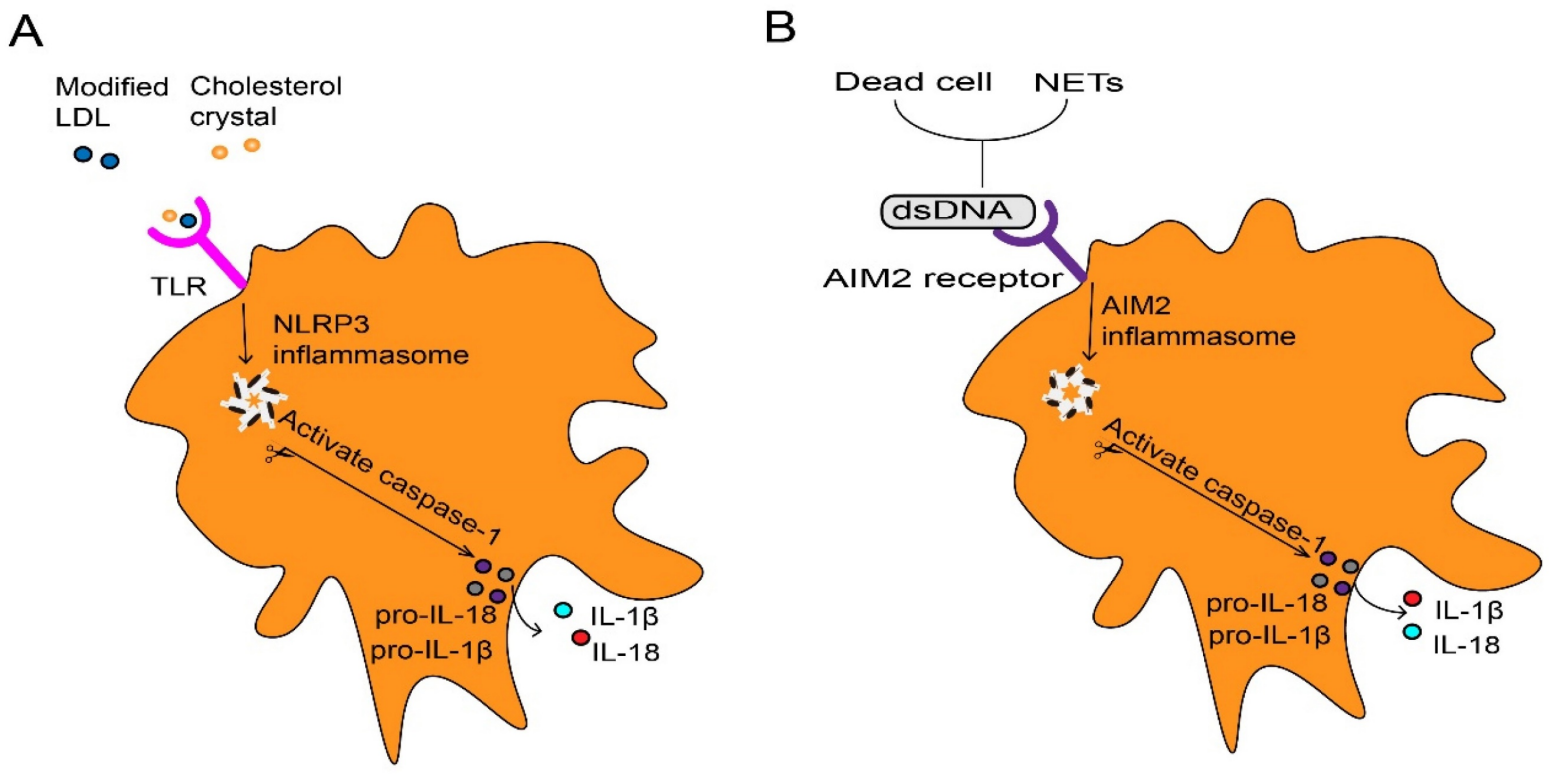
** Inflammasome activation in macrophage.** (A) Extracellular inflammatory stimuli such as modified LDL and cholesterol crystals, which are recognized by macrophages through TLR, thus promote transcription of pro-IL-1β and pro-IL-18 as well as NLRP3. Assembly of the NLRP3 inflammasome stimulates caspase-1 activation, which processes pro-IL-1β and pro-IL-18 into mature IL-1β and IL-18. (B) Dead cells and NETs release dsDNA as danger signals which lead to the AIM2 receptor activation. Activated AIM2 inflammasome also contributes to the maturation and production of IL-1β and Il-18 through activating protease caspase-1. LDL, low-density lipoproteins. IL, interleukin. TLR, toll-like receptor. NLRP3, NOD-like receptor protein 3. NETs, neutrophils extracellular traps. dsDNA, double-stranded DNA. AIM2, absent in melanoma.

These contribute as the majority of foam cells in atherosclerosis 31. The formation of foam cells is a key event, and they are trapped within the injured endothelium to form the first sign structure of atherosclerosis called fatty streak 21. At first, foam macrophages rather inhibit inflammatory responses 32. However, accumulation of modified lipoproteins result in oxidative stress then disrupts cellular metabolism and eventually leads to the death of foam cells 33. Dead foam cells are initially engulfed and cleared by macrophages through effervescence 34, but insufficient clearance results in the formation of a necrotic core of dead cells 21. Cell debris and modified LDL serve as danger signals that further promote the recruitment of atherogenic immune cells in plaques 21. Furthermore, inflammatory macrophages abundantly promote the NOD-like receptor protein 3 (NLRP3) inflammasome formation in response to modified LDL and cholesterol crystals in atherosclerotic lesions 35. This can further stimulate caspase-1 expression and drive immature interleukin 1β (IL-1β) and IL-18 into pro-inflammatory isoforms with strong pro-atherosclerotic effects (Fig. 2A) 33. IL-1 family cytokines not only increase oxidative stress in macrophages, but also promotes proliferation of them during atherosclerosis 36. The dead cells that release nuclear double-stranded (ds) DNA, partly derived from neutrophil extracellular traps (NETs) 37, act as cytosolic DNA that recognized by the absent in melanoma 2 (AIM2) receptor 38, which is restricted in macrophages 37. The recognition stimulates the activation of AIM2 inflammasome in macrophages, leading to the production of IL-1β and IL-18 in atherosclerosis (Fig. 2B). In the later stage of atherosclerosis, AIM2 macrophages are found surrounding the necrotic core 39. Targeting depletion of AIM2 significantly inhibited inflammatory macrophage proliferation and necrotic core formation 36 while promoting plaque stability by attenuating the death of SMCs 37. Additionally, macrophages within the plaque contribute to the expansion of the necrotic core, thereby increasing plaque instability. They achieve this by degrading extracellular matrix components through the release of Matrix metalloproteinases (MMPs), consequently thinning the fibrous cap 40. Macrophages contribute to plaque instability by providing tissue factors that induce bleeding within the plaque 41. Furthermore, the IL-17A/IL-17RA axis also increases aortic arch inflammation during atherogenesis through the induction of aortic chemokines, and the acceleration of neutrophil and monocyte recruitment to this site 42.

Different macrophage subsets exhibit distinct phenotypes and effects within atherosclerotic plaques Their plasticity is contingent upon the local microenvironment of the lesion, the macrophage origin, and the activation of intracellular signaling pathways 43. Single-cell sequencing of atherosclerotic aortas reveals that pathological macrophages cannot be simply classified into M1 and M2 44. Recent characterization of human coronary atherosclerotic lesions using single-cell RNA sequencing unveiled three distinct subpopulations of myeloid macrophages (My.0, My.1, and My.2) were present within plaques associated with major adverse cardiovascular diseases 45. Consistent with a previous study on macrophages in carotid plaques 46, My.0 and My.2 macrophages release various pro-inflammatory cytokines, detecting danger-associated molecular patterns (DAMPs) through toll-like receptors (TLR) 45. These inflammatory macrophages are major drivers of atherosclerosis and do not exist in healthy arteries 43. My.0 selectively expresses TNF, whereas My.2 macrophages are characterized by a selectively high level of CXCL3 expression and a tendency to encode IL-1β 45. My.1 macrophages mainly consist of foamy macrophages without inflammatory cytokines. They significantly express triggering receptors encoded on myeloid cells 2 (TREM2), which regulate lipid metabolism and cholesterol efflux 20. Furthermore, My.1 macrophages promote plaque stability by inducing fibrosis 20. The number of these macrophages decreases in symptomatic patients compared to asymptomatic patients 46.

### Neutrophils

**Figure 3:**
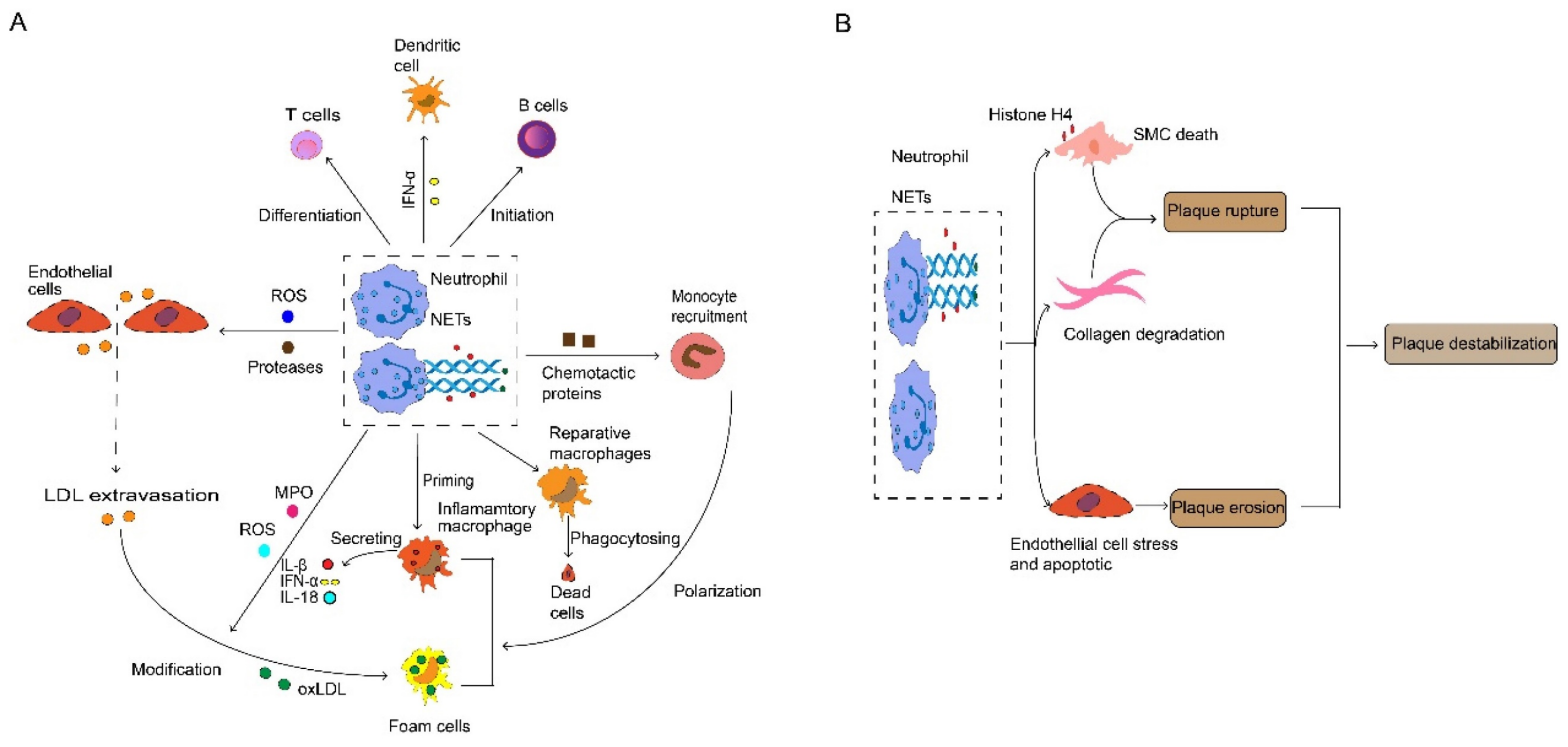
** Neutrophil and neutrophil extracellular traps (NETs) in atherosclerosis.** (A) During atherosclerosis progression, activated neutrophils release ROS and protease promotes endothelial cell stress and apoptosis, thus paving the way for myeloid cell accumulation and LDL extravasation. Neutrophils release various chemotactic proteins that contribute to monocyte recruitment. Consequently, infiltrated monocytes give rise to macrophage, neutrophil-derived ROS and MPO, which mediate modification of LDL, thereby promoting foam cell formation. Neutrophils and NETs also have an important role in the priming and activation of inflammatory macrophage, and stimulating the initiation of B cells and the differentiation of T cells, which triggers the generation and release of IL-1β, IL-18 and IFN-α. NETs protein-DNA complex serves as autoantigens to promote a strong IFN-α signal in dendritic cells. NETs are also involved in anti-inflammatory by activating macrophages to phagocyte dead cells. (B) Plaque destabilization can be divided into plaque rupture and plaque erosion, and neutrophil is the key element in the process. NETs containing cytotoxic histone H4 lead to death and lysis in SMCs, and neutrophils implicit in collagen degradation, thus provoking plaque rupture. Interestingly, when plaques are not at risk of rupture, neutrophils and NETs cause endothelial cell death and desquamation that contribute to plaque erosion. ROS, reactive oxygen species. LDL, low-density lipoproteins. MPO, myeloperoxidase. IL, interleukin. IFN-α, interferon-alpha. NETs, neutrophils extracellular traps. SMCs, smooth muscle cells.

Neutrophils are the first white blood cells recruited to the site of arterial damage 47. They induce strong inflammatory responses through various killing mechanisms 9. Neutrophils play a crucial role in the onset and progression of atherosclerosis 9, and their recruitment is necessary for the atherogenic effects caused by endothelium breaches 48. Significant accumulation of neutrophils is found in the intima of human coronary arteries 9, and neutralizing neutrophils can reduce arterial intimal thickening in mice under flow disturbance 48. Infiltrated neutrophils aggravate endothelium damage by secreting reactive oxygen species (ROS) and proteases (Fig. 3A), which contribute to the foundation of necrotic core formation by increasing the accumulation of leukocytes and LDL in the sub-endothelial region 9. A recent study showed that the association between neutrophil count and microvascular obstruction has been eliminated in AMI (acute myocardial infarction) patients treated with metoprolol and metoprolol inhibits neutrophil migration as a potential target for the therapeutic reduction of infarct size 49. In addition, neutrophils release various chemotactic proteins such as CCL2, Cathepsin G and α-Defensin. These chemokines accelerate the infiltration of monocytes 12, thus paving the way for macrophage expansion 15. Furthermore, the alarmins produced by neutrophils increase circulating monocytes by inducing the activation of bone marrow monocytes 50.

Neutrophils and their secreted products stimulate macrophages and promote the formation of foam cells. Catherine and α-defensin drives macrophages to polarize into an inflammatory state 9. Neutrophils produce reactive oxygen species (ROS) and myeloperoxidase (MPO), contributing to LDL modification, thereby accelerating the transition of macrophages into foam cells 9. Moreover, neutrophils inhibit the increase in macrophage effervescence, thereby exacerbating pathological inflammation 51. NETs released by neutrophils are characterized by reticular structure and contain de-concentrated chromatin and granular protein. These proteins are related to pathogen eradication, autoimmune diseases and vascular disease 52. An imbalanced immune response may lead to dysfunction of NETs and leakage of antimicrobial components, thereby exacerbating inflammation and host tissue damage 53. The contribution of neutrophils was demonstrated by targeted depletion using antibodies. Depletion of neutrophils results in decreased size of atherosclerotic lesions in mice 54. After sensing cholesterol crystals, NETs activate macrophages to activate NLRP3 inflammasome which eventually increases IL-1β generation 55. Conversely, the activation of NLRP3 in macrophages increases the accumulation of neutrophils and the formation of NETs in plaques 56. As mentioned above, NETs stimulate AIM2 inflammasome and increases caspase-1 expression in macrophages, thereby increasing IL-1β secretion 38. On the other hand, NETs can also directly enhance the TLR9/NF-kB (nuclear factor-k-gene binding) pathway in macrophages, promoting the production of IL-8 57 and Tumor Necrosis Factor-α (TNF-α) 58.

In addition to their effect on innate immune responses, neutrophils also have the ability to mediate adaptive immunity in atherosclerosis (Fig. 3A). Neutrophils can act as APCs that express major histocompatibility complex Ⅱ and costimulatory molecules in the cell surface after being stimulated by interferon-γ (IFN-γ). IFN-γ originate from various immune cells including itself 59, which induces T cell differentiation 47. For example, neutrophils facilitate the differentiation of CD4 T cells into Th1 and Th17 cells 59. Particularly, NETs can directly activate immature T cells, inducing Th17 differentiation 60, and further recruit more immune cells into the plaque via by promoting the activation of Th17 cells 55. Also, neutrophils play an important role in antigen presentation and the development of effector T cells through IFN-γ secretion 47. Neutrophil-derived B cell activating factor and a proliferation-inducing ligand affect the survival and activation of B cells 61. During myocardial infarction, recruited neutrophils release various alarmins which induce granulocyte formation to aggravate damage 62. They also promote mature B cells into plasma cells in the blood, which in turn accelerates atherosclerosis 63. The presence of long-lived antibody secreting cells may explain the risk of recurrence of myocardial infarction and stroke following the initial myocardial infarction. In addition, NETs stimulate the production of IFN-α in plasmacytoid dendritic cells to promote plaque growth 64.

The destruction of atherosclerotic plaque is a major driving factor of cardiovascular disease 65. Such destruction can be categorized into two different ways: plaque rupture, caused by the expansion of necrotic core, and plaque erosion, which means the thinning of fibrous cap 66. Neutrophils play an indispensable role in all forms of plaque instability (Fig. 3B). Compared to plaque erosion, plaque rupture usually accompanies a more severe inflammatory state, characterized by extremely thin fiber caps and higher lipid load 66. Neutrophils not only contribute to necrotic core expansion 52 but also increase lipid infiltration by disrupting the artery wall 9. Moreover, pathological SMCs attract NETs which release histone H4, thus thinning the fibrous cap by driving the death of SMCs 67. Neutrophils can also promote collagen degradation and accelerate plaque rupture 68. Due to the progress in lifestyle modifications and medical interventions, the incidence of plaque rupture and plaque superficial erosion-induced acute coronary artery diseases are rising 69. Plaque erosion is characterized by the preservation of vascular integrity and platelet rich thrombus. This mainly occurs in atherosclerosis lacking a lipid core or a compromised fiber cap, representing lower risk 70. Eroded plaques remain intact and are seen in sites of the vasculature where endothelial cells are deficient and disturbed flow occurs 71, facilitated by neutrophils and NETs that accelerate endothelial cell death and detachment 72. Furthermore, NETs not only promote platelet activation when binding to the injured endothelium 73 but also interact with locally activated platelets, initiating the coagulation cascade and accelerating the formation of wall thrombus, which consequently contributes to cardiovascular diseases 74.

Neutrophils mainly exert a pro-inflammatory response, but can also be anti-inflammatory 75-78. Neutrophils expressed high pro-inflammatory markers at Day 1 and high anti-inflammatory markers at Days 5 and 7 post-MI (myocardial infarction). Pro-inflammatory N1 neutrophils always dominate and can be mediated by DAMPs, and the percentage of anti-inflammatory N2 neutrophils gradually increased post-MI from Day 1 to Day 7 in mice 75. Neutrophils are also involved in the modulation of the healing and remodeling response: the protein S100A8/A9 (S100 calcium-binding protein A8/A9) in NETs activated macrophages to phagocyte dead cells 76. The transformation of neutrophils from pro-inflammatory to anti-inflammatory initiates the reparative process mostly by dedifferentiating cardiomyocytes and promoting the accumulation of reparative macrophages 77, 78.

### Dendritic cells

Dendritic cells (DCs) are another important coordination factor of immune-inflammatory response in atherosclerosis 10. DCs originate from hematopoietic progenitor cells, possess various pathogen recognition receptors (PPRs) and establish a network of sentinel cells on both the outer and inner surfaces of most tissues 10. While only a small number of DCs are present in the heart of healthy individuals, arteries susceptible to atherosclerosis harbor myeloid cells displaying characteristics and markers associated with DCs 79. The intimal resident DCs contribute to the retention of lipids in the endothelium during the initiation of atherosclerosis 80. Additionally, DCs and macrophages share similar functional characteristics 8, contributing to foam cell formation and accelerating necrotic core expansion 80, 81. Moreover, as the most potent and versatile antigen-presenting cells (APCs), DCs serve as an important bridge linking innate and adaptive immune responses 10. These cells absorb their self and foreign antigens through specialized surface receptors 10 and subsequently present antigens on major histocompatibility complex (MHC) molecules in the cell surface following danger signals 82. Upon activation, DCs migrate into the lymph node located below advanced plaques. There, the activated DCs physically interact with immature T cells, presenting antigens to initiate adaptive immunity 5. Such process is also observed in plaques 83.

DCs exhibit similar transcriptional characteristics and developmental potential in humans and mice 84. They can be widely divided into two main subtypes: conventional DCs (cDCs) and plasmacytoid DCs (pDCs) 10. pDCs, usually derived from lymphocytes and partly from monocytes, are characterized by large amounts of IFNs 64. These IFNs, for instance IFN-α, mediate the formation of atherogenesis through promoting maturation of cDCs 85, expansion of CD8^+^ cytotoxic T cells (CTLs) 86 and polarization of CD4^+^ T cells into T-helper 1 (Th1) cells 87. Moreover, pDCs enhance CD4^+^ T cells immunity through MHC class Ⅱ-restricted antigen presentation without IFN-α production 88. Typically found in the shoulder regions of human atherosclerotic plaques, increased plaque burden ultimately contributes to plaque rupture 89. Interestingly, pDCs exhibit both pro-atherogenic 90 and anti-atherogenic effects in mice 91. Such contradictory results may be due to the interaction with T cells and variations in animal models of atherosclerosis. DCs consist of type 1 conventional dendritic cells (cDC1s) and type 2 conventional dendritic cells (cDC2s) 10, serving as the main activator of the naive T cell pool 92. cDC1s mainly present exogenous antigens through MHC class Ⅰ, a process called cross-presentation, which is critical to the initiation of CD8^+^ CTLs 93. On the other hand, cDC2s are involved in the initiation of T cell responses 94. There are significant interactions between cDCs and CD4^+^ T cells in the aortic wall, promoting T cell activation, proliferation, and the production of IFN-γ and TNF-α cytokines 95. These proinflammatory cytokines increase the uptake of LDL by macrophages and the infiltration of immune cells 96, indicating that cDCs accelerate atherosclerosis by increasing chronic inflammation and formation of foam cells 95. Moreover, these cDCs exhibit interactions with antigen-experienced effector memory T cells within plaques, suggesting a recall reaction in the progression of atherosclerosis 95. The maturation and migration of DCs play a crucial role in facilitating interactions between T cells and DCs, forming the core of adaptive immunity. In the presence of CCR7, the receptor for CCL19 and CCL21, DCs migrate from peripheral tissues to draining lymph nodes 97. Additionally, circulating DCs exhibit a preference for migrating from the bloodstream to developing plaques or lymphoid tissues 10. Thus, decreased number of circulating pDCs can be used as a predictor of coronary artery disease 98. During atherosclerosis regression in mice, DCs migrate from atherosclerotic lesions into lymph nodes and circulation system 99. MyD88 is an adapter for downstream signal transduction through TLRs in mature DCs 100, and its depletion reduces the homing of effector T cells and Treg cells to plaque in atherosclerotic mice 101. Additionally, mediators derived from DCs play a significant role in adaptive immunity. For example, DCs release CCL17, inhibiting the expansion of regulatory T (Treg) cells in atherosclerosis and thereby promoting the recruitment of inflammatory monocytes 102. Also, the expression of CD40 on the cell surface by DCs contributes to Th1 polarization 103. Anti- and pro-atherosclerotic outcomes depend on interactions between T cells and DCs, followed by subsequent adaptive immune responses.

## Adaptive immunity

**Figure 4:**
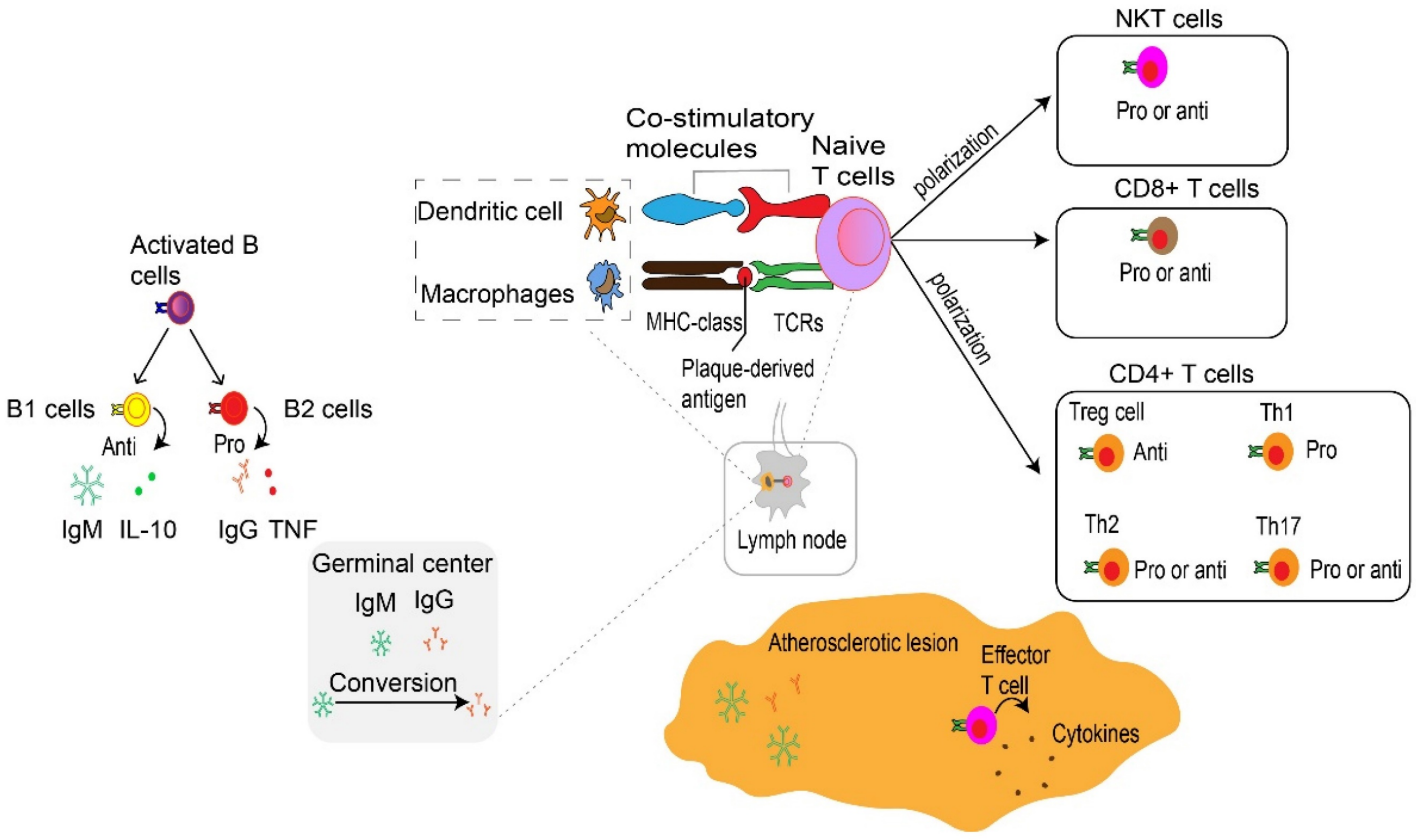
** Adaptive immune cells in atherosclerosis.** At the immune synapse, macrophages, dendritic cells and B cells, present lipid antigens to natural killer T (NKT) cells and peptide antigens to T cells, the latter engaging adaptive T cell and B cell responses. These antigens are macrophages and dendritic cells present plaque-derived antigens through MHC class molecules recognized by T cells through TCRs to naive CD4^+^ and CD8^+^ T cells in lymph nodes and atherosclerotic plaques, co-stimulatory molecules are also involved in the process which prime naive T cells and promote their proliferation and polarization. These matured effector T cells including CD8^+^ cells, CD4^+^ T cells such as Th1 cells, Th2 cells and Th17 cells as well as Treg cells together into the systemic circulation migrate to atherosclerotic lesions where they release various cytokines to promote atherogenesis or depress atherogenesis. CD8+ T cells in atherosclerotic lesions have also been found to have dual functions, with pro-atherogenic effects mediated by IFNγ production and macrophage activation, and atheroprotective effects via limiting the accumulation of macrophages and Th1 cells and B cell modulation. Th1 cells produce TNF-α and IFN-γ, indicating a pro-inflammatory and pro-atherogenic role of Th1 cells. Th2 and Th17 cells play a controversial role in atherosclerosis. Treg cells promote inflammatory resolution and dampen atherosclerosis progression via the production of IL-10 and TGFβ. The effects of NKT cells on the development of atherosclerotic plaques seem to be conflicting and need to be further investigated. Activated B cells mature into antibody-secreting plasma cells, and produce LDL-specific IgM and IgG antibodies that are enriched in atherosclerotic lesions. Conversion of IgM to IgG occurs in the germinal center located in the lymph node and form by B cells and Tfh cells. In addition, they secret IL-10 and TNF to regulate atherosclerosis progression. NKT, natural killer T cells. MHC, major histocompatibility complex. TCRs, specific T cell receptors. Th, T helper cells. Treg, regulatory T cells. IFNγ, interferon-gamma. TNF-α, Tumor Necrosis Factor-α. IL, interleukin. TGFβ, transforming growth factor β. LDL, low-density lipoproteins. Ig, immunoglobulin. Tfh, T follicular cells. Pro, Pro-inflammatory. Anti, Anti-inflammatory.

**Figure 5:**
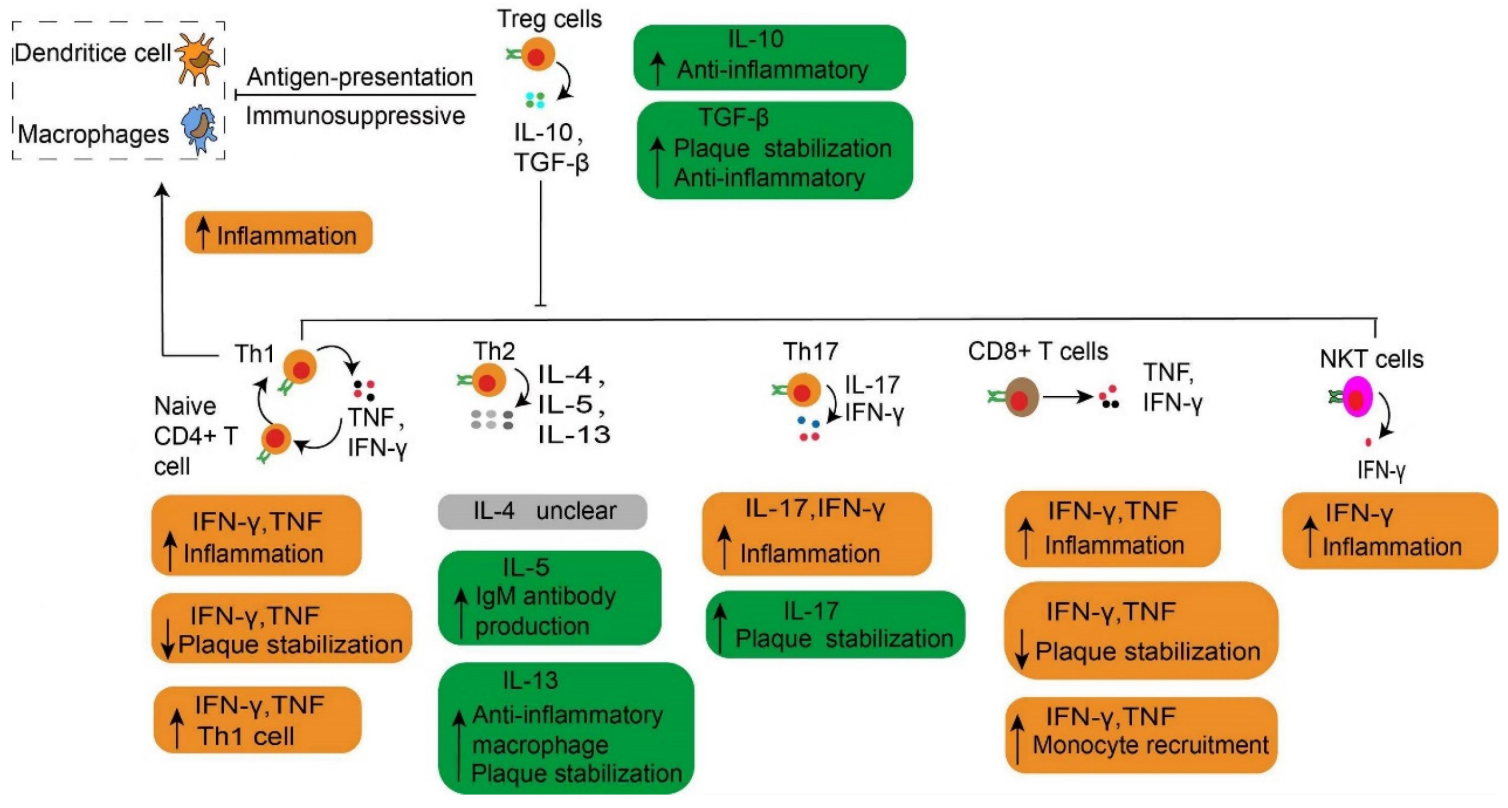
** The main cytokines released by each effector T cell in atherosclerosis.** The main cytokines released by each effector T cell are described, together with their roles in atherosclerosis. Especially, the Treg cells play an immunosuppressive effect by depressing the antigen-presentation process. The Th1 cells secrete TNF and IFN-γ, which increase inflammation by contributing to the transformation of naive CD4+ T cells to Th1 cells. NKT cells have a pro-atherogenic effect due to producing a high amount of pro-inflammatory cytokines such as IFNγ. Treg, regulatory T cells. Th, T helper cells. IFNγ, interferon-gamma. TNF, Tumor Necrosis Factor. NKT, natural killer T cells.

T cells, B cells, and their secondary products are the main components of adaptive immunity. The adaptive immune system coordinates the formation of atherosclerosis and the stability of plaque (Fig. 4 and 5). Adaptive immunity is primarily controlled by antigen presentation. In atherosclerosis, antigen-presenting cells (APCs) such as macrophages within atherosclerotic plaques, adventitial B cells, and DCs present peptide antigens. These antigens are recognized by T cells through specific T cell receptors (TCRs) 96. During this process, APCs present plaque-derived peptides on their MHC (major histocompatibility complex) class Ⅰ or MHC class Ⅱ molecules to immature CD4^+^ or CD8^+^ T cells, respectively 97. One finding indicate that the PD-1/PD-L (programmed death-1, Programmed death-ligand 1) pathway plays an important role in down-regulation of pro-atherogenic T cell response and atherosclerosis by limiting APC-dependent T cell activation 104. APCs released co-stimulatory molecules simultaneously bind to TCR, inducing not only clonal proliferation of T cells 105 but also occupying a dominant position in differentiation into effector T cells 96. The cytokines secreted by APCs also affect T cell differentiation 106. For example, IL-12 released by APCs contributes to the maturation of pro-inflammatory T helper type 1 (Th1) cells 107, TGF-β promotes transformation of CD4^+^ T cells into CD4^+^ regulatory T cells 108. IL-2 induced immature CD4^+^ T cells to transform into regulatory T cells (Tregs) 109. MHC class I and II molecules, along with co-stimulatory molecules provided by APCs, establish the basis of adaptive immunity in atherosclerosis 110. Results from TCR sequencing have demonstrated oligoclonal amplification of activated T cells, indicating their status as antigen-experienced effector T cells 111. These effector cells, with specific antigen memory, migrate from draining lymph nodes to the systemic circulation and accumulate in atherosclerotic plaques 5. Notably, T cells experiencing antigen show memory response upon re-stimulation 43. In contrast to innate immunity, adaptive immunity is a rather slower process, but is highly specific and persistent. Such characteristic is largely due to the development of memory effector cells. Similar to macrophages, T cells are found in all stages of atherosclerosis 112, with activated T cells as a sign of atherogenesis 5. Atherosclerotic lesions containing T cells and are often enriched in LDL-specific antibodies 92. Blocking the co-stimulatory pathway of T cells may be a therapeutic target for CVDs. On the contrary, inhibiting the T cell co-inhibitory pathway and stimulating the co-stimulatory pathway may pose a serious risk of cardiovascular events 113.

Both CD4^+^ and CD8^+^ T cells are found in human aortas 46, and particularly CD8^+^ T cells are prevalent in humans 114 and murine plaques 115. On the other hand, CD4^+^ T cells are the key regulator of adaptive immunity in atherosclerosis. They facilitate B cells to produce antibodies 97 and support CD8^+^ T cells to exert the full potential of their cytotoxic effects 43. After activation, CD4^+^ T cells mainly differentiate into distinct T-helper (Th) subtypes of Th-1, -2, -17, follicular helper T cells or Treg cells 97. These Th cells or Treg cells, along with their products, respectively contribute to immune activation or immunosuppression in atherosclerosis. Upon activation, these effectors use CCR5 116 and CXCR6 117 to home into atherosclerotic lesions. Then they target the primary ligand of CCR5 provided by activated platelets to deplete CCL5 118 or block the interaction of CCL5 and CCR5, thereby reducing the infiltration of CD4^+^ T cells into advanced plaques 116. However, CCR5 deficiency does not affect the early stages of atherosclerosis 119. This observation suggests that Treg cells might neutralize the pro-atherosclerotic effects of Th cells. It is generally accepted that Treg cells play a dominant role in the initial stage of atherosclerosis by limiting the formation of inflammation and the development of atherosclerosis. Here, we discuss the most prevalent subtypes of CD4^+^ T cells and their pivotal role during the progression of atherosclerosis. The role of CD8^+^ T cells and B cells related to atherosclerosis is also discussed.

### Th1 cells

Atherosclerosis is known to be a Th1-related disease. Th1 cells are the most abundant and predominant CD4^+^ effector T cell subtypes in atherosclerotic plaques 97, 120. Th1 cells are characterized by expression of the defining T box transcription factor (T-bet) 121. They also release pro-inflammatory cytokines IFN-γ and TNF within lesions 122, which can induce the activation of macrophages and T cells, amplifying atherogenic inflammation 96. Several experimental studies have demonstrated that Th1 inhibition via genetic deficiency of T-bet or IFN-γ, or by deleting its receptor, significantly protects mice from the development of atherosclerosis 96. Th1-derived IFN-γ induces plaque instability by restraining the proliferation of vascular smooth muscle cells (VSMCs) 123 and accelerating the degradation of collagen matrix 124. TNF-α, mainly released by T cells, contributes to increased lesion size by stimulating the infiltration of leukocytes 125 and LDL 126. Surprisingly, the elevated level of IFN-γ produced by Th1 cells, which synergize with IL-12 to promote CD4^+^ T cells to skew towards Th1 127. TNF-α is not just an effector but also an initiator of inflammatory Th differentiation 128.

### Th2 cells

Th2 cells play a controversial role in atherosclerosis 43. CD4^+^ T cells express the transcription factor GATA binding protein 3 (GATA3) within the Th2 subtype, which is important for humoral immunity 129. Th2 cells predominantly produce IL-4, IL-5, and IL-13 in plaques 130. IL-4 promotes atherogenesis 131, therefore targeted depletion of IL-4 inhibits atherogenesis in mice 132. However, IL-4 levels are also negatively correlated with clinical atherosclerosis in humans 133. Even more, exogenous IL-4 does not drive the expansion of lesions in mice 134. In contrast to IL-4, Th2 -derived IL-5 and IL-13 antagonize the Th1 response and show a protective effect in atherosclerosis 135. IL-5 promotes the production of oxLDL-targeted antibodies by inducing B1 cell maturation, thereby suppressing inflammation and the formation of necrotic cores through the elimination of dead cells 136. Administration of IL-13 in atherosclerosis models has been shown to stabilize atherosclerotic plaques via inducing collagen expression 97 and inhibiting monocyte/macrophage infiltration 137.

### Th17 cells

Th17 cells are the third largest subpopulation of CD4^+^ T cells in atherosclerosis 97. Th17 cells are characterized by the expression of transcription factor RAR-related orphan receptor gamma (RORγ) and are activated by IL-23 138, acting as the main producer of IL-17 139. The plasticity of Th17 after recognizing its own antigen is similar to Tregs 97. It depends on local inflammatory environment 140 and the synergistic regulation of Th17 differentiation by IL-6 and transforming growth factor-β (TGF-β) 138. In the presence of IL-6 and IL-β, Th17 cells release IL-17 and IFN-γ to accelerate atherosclerosis progression 141. In addition, pathogenic Th17 cells secrete IL-6 and granulocyte-macrophage colony-stimulating factors (GM-CSFs) 139, stimulating leukocyte accumulation in plaques 142. Pro-inflammatory Th17 cells aggravate the development of atherosclerotic plaque in mice 143. Inhibition of IL-17 is shown to alleviate the development of atherosclerosis 142. On the other hand, Th17 enhances collagen production released by SMCs which increases plaque stability 144, so blockade of IL-17 leads to plaque fragility 145. This potentially reduces the TGF-β generation, which is dependent on the IL-17 pathway 146.

### Treg cells

**Figure 6:**
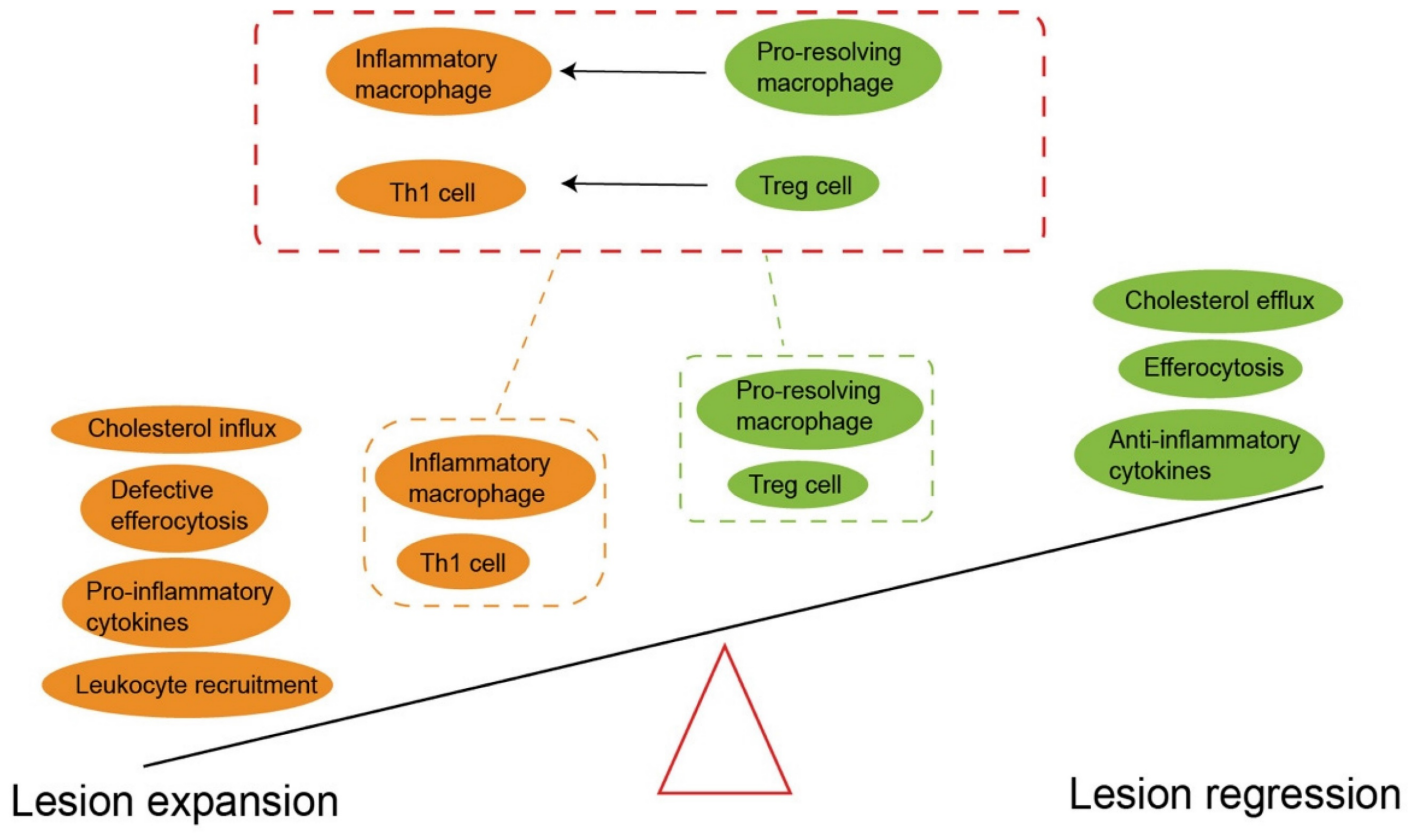
** The imbalance between pro-inflammatory and anti-inflammatory signals drives atherosclerosis.** The prevailing notion is that Treg predominates before disease onset. However, the imbalance of the pro-atherogenic effect (yellow) and anti-atherogenic effect (green) promotes atherogenesis. It should be noted Treg cell plasticity and instability mainly converts to Th1 cells in plaque formation. The pro-resolving macrophage also can polarize inflammatory macrophage to accelerate atherosclerosis progression. An increased anti-atherogenic effect may contribute to atherosclerosis regression. Treg, regulatory T cells. Th1, T helper 1 cells.

The Treg cell subset is CD25 positive and expresses the defined forkhead box protein P3 (Foxp3) transcription factor as a specific marker for human Treg cells, used to distinguish Treg from non-regulatory T cells 97, 147. Foxp3 controls Treg differentiation and plays a vital role in both the function and activation of Foxp3-expressing T cells 147. Treg cells exhibit inhibitory activity against various immune cells, including the proliferation and functional activity of CD4+ and CD8+ T cells, B cells, APCs, NKT, and cytokine production 147. Treg cells release anti-inflammatory cytokines such as TGF-β and IL-10, and thus protects from inflammation and subsequent atherosclerosis in humans and mice 43. Treg cells are important immunosuppressive modulators which collaborate with TGF-β and IL-10 to inhibit the proliferation and activation of pro-inflammatory effector T cells 148. They also suppress antigen presentation and reduce plaque inflammation 43. Notably, Treg depletion leads to a significant increase in lesions which is independent of vascular inflammation in atherosclerosis models 149. In addition, TGF-β derived from CD25^+^ Foxp3^+^ Treg cells contributes to plaque stability by increasing collagen contents 5. The progression of atherosclerosis is mainly determined by the balance between Treg cells and pro-inflammatory T cells, especially Th1 cells (Fig. 6). In the early stages of atherosclerosis, Th1 cells and monocyte-derived macrophages form atherosclerotic lesions 5. Treg cells counteract against these inflammatory cells and suppress the growth of lesions 150. However, inflammatory CD4^+^ effector T cells increase and Treg cells decrease in lesions, and thus disrupt the balance of these two, accelerating the progression of atherosclerosis 150.

Treg cells are important linkers between immunity and lipoprotein metabolism 43. Reduced Treg cells lead to an impaired ability to regulate very low-density lipoprotein (VLDL) and LDL in the blood, and partly aggravates hypercholesterolemia 149. Conversely, long-term hypercholesterolemia inhibits the tolerance and accumulation of Treg cells, and also enhances the potential of inflammatory T cells to counteract Treg cells 151. Additionally, accumulation of cholesterol crystals amplifies the activation of immune cells and therefore induces complex inflammatory responses 55. Surprisingly, under atherosclerosis-related inflammation, Treg cells lose the expression of CD25 and Foxp3. Instead, they show high plasticity (Fig. 6), expressing other effector T-specific transcription factors, chemokine receptors and cytokines 97. Single cell RNA sequencing studies have demonstrated that Foxp3^+^ Treg cells transform into Th1 cells and begin expressing IFN-γ in advanced plaques 152. In addition, a subset of unstable Treg cells acquire the characteristics of T-follicle helper cells (Tfh) or convert into ex-Treg cells, both phenotypes losing the immunosuppressive ability during the progression of atherosclerosis 153.

In addition to suppressing the progression of atherosclerosis, Treg cells promote plaque regression by resolving inflammation and repairing damaged tissues 22. In the presence of an LDL reduction strategy, the amount of Treg cells in plaques should not decrease 150. Expansion of Treg cells is considered as a sign of regression of atherosclerosis 22, and these Treg cells originate from peripheral immature T cells rather than thymocytes 154. Treg cells not only inhibit continuously activated macrophages and inflammatory effector T cells, but also promote macrophage polarization into M2 macrophages in degraded plaques 22. This M2 polarization increases the clearance of dead cells, reduces necrotic cores and lead to regression of inflammation 22. Activated M2 macrophages express IL-10 and TGF-β, which in turn promote Treg cell expansion 155.

### CD8^+^ T cells

MHC class I molecules present apolipoprotein B-derived epitopes to CD8^+^ T cells 97. Following stimulation, CD8^+^ T cells polarize into CD8^+^ cytotoxic T lymphocytes (CTLs), possessing the ability to eliminate viral infections and other abnormal cells through various cytotoxic pathways 97. CD8^+^ T cells are reported to exert dual roles in atherosclerosis: they can both promote atherosclerosis or protect from its development in murine models 115, 156. CD8^+^ CTLs utilize cytotoxic granules, particularly perforin-1 and granzymes, to promote the formation of necrotic cores by inducing apoptosis of macrophages, VSMCs and endothelial cells. Additionally, CD8^+^ T cells aggravate inflammation and monocytosis in the bone marrow through TNF-α 115 and IFN-γ production 157. Of note, perforin-1 and granzymes released by CD8^+^ CTLs exhibit inhibitory actions against APCs and other effector T cells, potentially impeding the onset of atherogenesis. A subset of CD8^+^ T cells shows regulatory functions that actively inhibit atherosclerosis by limiting the accumulation of macrophages and Th1 cells 158 and B cell-mediated atherogenic antibody production 156. In advanced human plaques, CD8^+^ T cells account for the majority of effector T cells 159 and are localized in predominantly fibrous cap sites 160. The increased blood CD8^+^ T cells is a commonly seen in patients who recently suffered from coronary artery diseases 161. Although CD8^+^ T cells can recognize apolipoprotein B-derived epitopes, their effect on atherosclerosis is not fully understood.

### Natural Killer T cells

Natural killer T (NKT) cells are another subset of T cells that express unique invariant T cell receptors and natural killer cell surface molecules, such as CD161 (also known as NK1.1 in mice) and killer cell immunoglobulin like receptors (similar to the Ly49 family in mice) 162. NKT cells have the ability to respond to lipid derived antigens presented by CD1d on antigen-presenting cells. Symptomatic plaques of carotid atherosclerosis are associated with increased NK cell infiltration and elevated serum levels of NK activated receptor ligands 163. In mice, NKT cells are considered atherogenic because they produce a large number of proinflammatory cytokines, such as IFNγ 164. In humans, the number of NKT cells in rupture-prone plaques is higher compared to stable plaques 164, but the exact mechanism of this process is still unclear. However, a recent study showed that the high activation or gene deletion of NKT cells would not affect the progression of atherosclerosis in the mouse model 165. In general, the effect of NKT cells on the development of atherosclerotic plaque seems to need further study. The adaptive immune system coordinates the formation of atherosclerosis and the stability of plaque (Fig. 4 and 5).

### B cells

B cells can accelerate or inhibit atherosclerosis, mainly by producing antibodies to induce regulatory T cell responses 92. Like T cells, B cells have specific B cell receptors (BCR) which recognize foreign and autoantigens. BCRs mediate the activation, development and differentiation of B cells in draining lymph nodes 92. Generally, the number of B cells present in atherosclerosis plaque is low, with most of them stay in the outer membrane with lymphatic structure 166. Upon activation, B cells mature into plasma cells and secrete antibodies; in particular LDL-specific antibodies. These antibodies obtain high affinity in the germinal center of the lymphoid nodes, and are maintained by B cells and Tfh cells 167. B cells can be subcategorized into B1 and B2 cells 92. B1 cells can independently produce antibodies without T cells 92, while B2 cells require Tfh cells for activation and transformation into B2 plasma cells that generate soluble antibodies in the blood 96. B1 cells can suppress the development of atherosclerosis by secreting natural immunoglobulin M (IgM) antibodies directly counteracting ox-LDL, thereby limiting the formation and activation of foam cells 92. B1 cells also have a regulatory effect by releasing IL-10 to modulate inflammation responses 168. On the other hand, IgG antibodies produced by B2 cells amplify inflammation reaction in atherosclerosis and contribute to the activation and polarization of innate immune cells 92. In addition, B cells can transform IgM antibodies into other isotypes such as IgG, further promoting the progression of atherosclerosis 167. The developing B cells can also polarize into other phenotypes such as innate response-activated B cells and cytokine-producing B cells, which increase Th1 cell immune response 169 and pro-inflammatory cytokines TNF-α 170, respectively.

## Conclusion

Atherosclerosis is a chronic inflammatory disease mainly driven by a maladaptive immune response. The immune system participates in all stages of atherosclerosis, from the initiation to progression, and also in atherosclerosis-associated complications. Emerging evidence suggests that combining LDL reduction strategies with anti-inflammatory drugs can successfully prevent major cardiovascular adverse events. However, systemic immune suppression can result in increased susceptibility of fatal infections. Therefore, greater attention should be directed toward safer and more precise therapeutic interventions targeting inflammation related to atherosclerosis. Especially, the subtypes of immune cells within plaques undergo phenotypic transformations under complex inflammatory conditions. Understanding the mechanisms underlying immune cells and their secondary products that orchestrate inflammation and atherogenesis would facilitate the development of a novel strategy. Such attempts should focus on maintaining the protective effect of the immune system while controlling maladaptive immune responses against the host.
